# Cannabis Use Reported by Patients Receiving Primary Care in a Large Health System

**DOI:** 10.1001/jamanetworkopen.2024.14809

**Published:** 2024-06-05

**Authors:** Lillian Gelberg, Dana Beck, Julia Koerber, Whitney N. Akabike, Lawrence Dardick, Clara Lin, Steve Shoptaw, Marjan Javanbakht

**Affiliations:** 1 Department of Family Medicine, UCLA David Geffen School of Medicine, Los Angeles, California; 2 Department of Health Policy and Management, UCLA Fielding School of Public Health, Los Angeles, California; 3 UCLA School of Nursing, Los Angeles, California; 4 Department of Epidemiology, UCLA Fielding School of Public Health, Los Angeles, California; 5 Department of Internal Medicine, UCLA David Geffen School of Medicine, Los Angeles, California

## Abstract

**Importance:**

Despite the changing legal status of cannabis and the potential impact on health, few health systems routinely screen for cannabis use, and data on the epidemiology of cannabis use, and especially medical cannabis use among primary care patients, are limited.

**Objective:**

To describe the prevalence of, factors associated with, and reasons for past–3 month cannabis use reported by primary care patients.

**Design, Setting, and Participants:**

This cross-sectional study used electronic health record data from patients aged 18 years and older who had an annual wellness visit between January 2021 and May 2023 from a primary care clinic within a university-based health system in Los Angeles, California.

**Exposures:**

Factors of interest included age, race and ethnicity, sex, employment status, and neighborhood Area Deprivation Index (ADI).

**Main Outcomes and Measures:**

Cannabis use was assessed using the Alcohol Substance Involvement Screening Test (ASSIST). Patients were also asked about reasons for use, symptoms for which they used cannabis, and mode of use.

**Results:**

Among the 175 734 patients screened, the median (range) age was 47 (18-102) years; 101 657 (58.0%) were female; 25 278 (15.7%) were Asian, 21 971 (13.7%) were Hispanic, and 51 063 (31.7%) were White. Cannabis use was reported by 29 898 (17.0%), with 10 360 (34.7%) having ASSIST scores indicative of moderate to high risk for cannabis use disorder (CUD). Prevalence of cannabis use was higher among male patients than female patients (14 939 [20.0%] vs 14 916 [14.7%]) and younger patients (18-29 years, 7592 [31.0%]; ≥60 years, 4200 [8.5%]), and lower among those who lived in the most disadvantaged neighborhoods (ADI decile 9-10, 189 [13.8%]; ADI decile 1-2, 12 431 [17.4%]). The most common modes of use included edibles (18 201 [61.6%]), smoking (15 256 [51.7%]), and vaporizing (8555 [29.0%]). While 4375 patients who reported using cannabis (15.6%) did so for medical reasons only, 21 986 patients (75.7%) reported using cannabis to manage symptoms including pain (9196 [31.7%]), stress (14 542 [50.2%]), and sleep (16 221 [56.0%]). The median (IQR) number of symptoms managed was 2 (1-4), which was higher among patients who were at moderate to high risk for CUD (4 [2-6] symptoms).

**Conclusions and Relevance:**

In this study, cannabis use and risk of CUD were common, and more than three-quarters of patients who reported any cannabis use reported doing so to manage a health-related symptom. These findings suggest that integration of information regarding cannabis use for symptom management could help provide a crucial point-of-care opportunity for clinicians to understand their patients’ risk for CUD.

## Key Points

**Question:**

What is the prevalence of past 3-month cannabis use, and what are the reasons for use among patients in a large health system?

**Findings:**

In this cross-sectional study of 175 734 patients, 17.0% reported cannabis use, among whom 34.7% had results indicative of moderate to high risk for cannabis use disorder. While most patients (76.1%) reported using cannabis to manage a health symptom, very few patients identified as medical cannabis users.

**Meaning:**

Given the high rates of cannabis use, especially for symptom management, and the high levels of disordered use, it is essential that health care systems implement routine screening of primary care patients.

## Teaser

This cross-sectional study evaluates how many patients report cannabis use in primary care wellness visits, what reasons they report for use, and what factors are associated with cannabis use.

## Introduction

Cannabis use is common and has become increasingly prevalent in the United States, with 45.7% of those surveyed as part of the 2021 National Survey on Drug Use and Health (NSDUH) reporting lifetime use, 18.7% past-year use, and 13.0% past-month use.^1^ Furthermore, between 2002 and 2014, past-month cannabis use among young people (aged 18 to 25 years) increased from 17.3% to 19.6%, increasing to 24.1% in 2021.^1,2^ Among NSDUH respondents aged 26 years and older, past-month cannabis use also increased from 4.0% in 2002 to 6.6% in 2014 and nearly doubled to 12.2% in 2021.^1,2^ These increases have been noted across most demographic subgroups, including sex, age, race and ethnicity, education, and region.^2,3,4,5,6^

Recent legislation in the United States has expanded access to cannabis, with 38 states, 3 territories, and the District of Columbia allowing for medical use and 24 of these states also permitting recreational use of cannabis (as of March 2024).^7^ The changing legal landscape has likely played a substantial role in shaping public perception, leading to a decrease in stigma and an increase in the perception that cannabis use involves no risk.^3,8,9^ Concurrently, there has been an increase in cannabis product potency (ie, higher tetrahydrocannabinol [THC] concentration), and greater variation in types of products (eg, flower products, extracts) and modes of use (eg, edibles, vaping).^10,11^

The 2020 US Preventive Services Task Force recommended that primary care clinicians screen adult patients for substance use, including cannabis.^12^ Valid, patient-reported cannabis screening tools are available, and screening for cannabis use in primary care is feasible, yet routine screening in primary care remains limited.^13,14,15^ One of the few health systems to implement routine cannabis screening found that as many as 22.0% of primary care patients reported past-year cannabis use.^16^ Furthermore, more than half the patients who reported cannabis use for medical reasons had conditions for which cannabis use might confer risks, highlighting the importance of cannabis screening in primary care settings. Additionally, to our knowledge, no health systems have yet implemented routine screening that examines patient-reported reasons for cannabis use, especially medical cannabis use. To bridge this gap, we report findings from a routine, patient-reported, cannabis screening program for primary care patients seeking care in a large health system. Built into this effort, we sought to identify reasons for use as well as differences in the prevalence of cannabis use and risk for cannabis use disorder (CUD) by age, sex, race and ethnicity, and socioeconomic status.

## Methods

This is a cross-sectional study of existing electronic health record (EHR) data collected between January 2021 and May 2023. This study followed the Strengthening the Reporting of Observational Studies in Epidemiology (STROBE) reporting guidelines. The project was approved by the institutional review board at the University of California, Los Angeles, and the need for informed consent was waived by the ethics committee because the research posed no more than minimal risk, the waiver would not adversely affect the rights and welfare of the participants, and the research could not be practicably carried out without a waiver.

### Study Setting and Design

The health care system that serves as the setting for this study is a large, university-based system in Los Angeles, California, and encompasses a geographically diverse area covering 2500 square miles, more than 200 clinics, and 4 hospitals.^17^ Starting in January 2021, EHR-based, automated, previsit, patient self-administered cannabis screening was implemented. Patients eligible for screening were (1) aged 18 years or older and (2) had a new visit or an annual wellness visit through one of the primary care clinics within the system. The Tobacco and Cannabis Questionnaire (TCQ; eAppendix in Supplement 1) is sent as part of a larger set of previsit health questionnaires using MyChart, a patient portal that offers patients access to portions of their EHR. At the time of this study, the patient portal as well as all the related functionality (such as the TCQ) was only available in English. For patients who did not complete their previsit questionnaires, the health system implemented a clinic workflow at visit registration to remind and assist patients to complete questionnaires prior to seeing their clinician. However, patients can opt out of completing the questionnaire. During the study period, there were 235 009 unique patients who had an eligible visit, among whom 175 734 (74.8%) completed the cannabis screening questionnaire. It is estimated that approximately 85% of the patient population actively uses the patient portal, meaning that of the 235 009 patients, only 200 000 would have received the screening questionnaire, resulting in a potential response rate of 87.8%.

### Data Collection and Measures

#### Sociodemographic Factors

Demographic characteristics were extracted from the EHR including age, race and ethnicity, sex, employment status, and sexual identity. Race and ethnicity are based on self-report with separate questions assessing race and ethnicity. Answer choices for ethnicity include (1) Hispanic, Latina, Latino, Latinx, or Spanish; (2) not Hispanic, Latina, Latino, Lantinx, or Spanish; and (3) choose not to answer. Answer choices for race include (1) Alaska Native or American Indian or Indigenous; (2) Asian; (3) Black or African American; (4) Middle Eastern or North African; (5) multiple races; (6) Native Hawaiian or Pacific Islander; (7) White; (8) other; (9) do not identify with a race; and (10) choose not to answer. Those who reported do not identify with any race, other, and multiple races were combined into a single category (other) for analysis. Unique to the EHR data are linkage of the patient’s zip code from their most recent address to the Area Deprivation Index (ADI). The ADI is a publicly available, census-based index constructed using 17 indicators to characterize socioeconomic disadvantage of a neighborhood.^18^ It has been validated and used in a number of studies to link neighborhood disadvantage to health behaviors and outcomes.^18,19,20,21,22^

#### Cannabis Use

Self-reported cannabis use (referred to as cannabis use hereafter) was based on the World Health Organization’s validated Alcohol Substance Involvement Screening Test (ASSIST) version 3.0,^23,24^ as modified by the National Institute of Drug Abuse.^25^ The ASSIST was designed to be used in health care settings and has been validated among diverse population groups; patient self-administration has high test-retest reliability.^26,27^ Patients are asked, “in the past 3 months, how often have you used cannabis?” with answer choices including never, once or twice, monthly, weekly, or daily/almost daily. Those who report no cannabis use in the past 3 months skip the subsequent 5 ASSIST questions, which identify risky use, including questions that assess desire to use cannabis, problems associated with cannabis use, and efforts to control or stop using. Real-time scoring of the ASSIST within the EHR system alerts clinicians of patients reporting risky cannabis use. In consultation with the ASSIST developers and motivated by a desire to identify patients who were not just frequent users of cannabis but also experiencing health or social consequences as a result of their cannabis use, the real-time scoring algorithm was modified, and the cutoff for moderate risk of CUD was increased from a score of 4 to a score of 8. Consequently, low risk for CUD was defined as a score of 7 or less (compared with ≤3 in the original scoring algorithm), moderate risk for CUD was defined as a score of 8 to 26 (compared with 4-26 in the original scoring algorithm), and we continued to define high risk of CUD based on a score of 27 or greater.^23,24^

Reasons for cannabis use, symptoms for which cannabis was used, and modalities of cannabis use were assessed using previously validated questions.^28,29,30^ Specifically, patients were asked whether they use cannabis for recreational reasons, medical reasons, or both. Regardless of their response, all patients were asked about symptoms for which they use cannabis, such as pain, sleep, depression, anxiety, and appetite. To assess the mode of cannabis use, patients are asked to select all applicable options from a list including (1) smoke, such as a joint, bong, blunt, spliff or pipe; (2) eat, such as brownies, cakes, cookies, candy, or pills; (3) drink, including tincture, tea, cola, or alcohol; (4) vaporize using a vaporizing device; (5) dab using waxes or concentrates in a dab rig or device; and (6) apply to skin using lotion, ointment, patch, or salve.

### Statistical Analysis

Outcomes of interest included any cannabis use in the past 3 months and being at moderate to high risk for CUD. Risk of CUD was only calculated for patients who reported cannabis use in the past 3 months; thus all analyses related to risk of CUD are limited to this subset of patients. Mode of cannabis use was also examined, including descriptive statistics for each specific mode as well as composite variables for any inhaled or ingested cannabis. Covariates of interest included age, sex, race and ethnicity, employment status, and neighborhood disadvantage (ADI). For this analysis, we used the ADI state ranking, which was categorized into deciles, with patients living in neighborhoods in the highest decile having the most disadvantage. Differences in the prevalence of these outcomes by sociodemographic characteristics were assessed using *t* tests, χ^2^ methods, and nonparametric tests as appropriate. All *P* values were from 2-sided tests, and comparisons were considered statistically meaningful if the 95% CIs did not overlap. Factors associated with cannabis use and risk for CUD were assessed using multivariable logistic regression analyses. Results were considered statistically significant when 95% CIs did not cross the null value of 1. All analyses were conducted using SAS version 9.4 (SAS Institute).

## Results

Among the 175 734 patients who completed cannabis screening, the median (range) age was 47 (18-102) years; more were female (101 657 [58.0%]), and the 3 largest racial and ethnic groups were Asian (25 278 [15.7%]), Hispanic (21 971 [13.7%]), and White (51 603 [31.7%]) (Table 1). Unemployment was reported by 22 006 patients (14.0%), with 1367 patients (1.1%) living in areas ranking in the highest deciles of the Area Deprivation Index (ie, decile 9-10).

**Table 1.** Sociodemographic Characteristics of Adult Primary Care Patients Screened for Cannabis Use, January 2021 to May 2023

Characteristic	Patients, No. (%) (N = 175 734)^a^
Age, y	
18-29	24 483 (13.9)
30-39	36 402 (20.7)
40-49	33 564 (19.1)
50-59	32 097 (18.3)
≥60	49 188 (28.0)
Sex	
Female	101 657 (58.0)
Male	73 929 (42.0)
Sexual minority^b^	11 688 (7.4)
Race and ethnicity	
American Indian or Alaska Native	312 (0.2)
Asian	25 278 (15.7)
Black or African American	7057 (4.4)
Hispanic	21 971 (13.7)
Middle Eastern or North African	4688 (2.9)
Other^c^	50 577 (31.4)
White	51 063 (31.7)
Unemployed	22 006 (14.0)
State ADI rank	
Decile 1-2	71 350 (56.0)
Decile 3-4	32 981 (25.9)
Decile 5-6	15 871 (12.4)
Decile 7-8	5912 (4.6)
Decile 9-10	1367 (1.1)

Abbreviation: ADI, Area Deprivation Index.

^a^ Sum may not equal total due to missing data, with missingness as follows: 0 for age; 148 (0.08%) for sex; 14 779 (8.4%) for race and ethnicity; 18 510 (10.5%) for employment; 17 300 (9.8%) for sexual identity; and 48 307 (27.5%) for state ADI rank.

^b^ Sexual minority includes those identifying as lesbian, gay, bisexual, or other sexual orientation.

^c^ Other race and ethnicity includes those who selected other, multiple races, or do not identify with a race.

### Prevalence of Cannabis Use and CUD

Overall, 29 898 patients (17.0%) reported cannabis use in the past 3 months, with a higher prevalence of cannabis use among male patients (14 939 [20.0%; 95% CI, 19.9%-20.5%]) compared with female patients (14 916 [14.7%; 95% CI, 14.5%-14.9%]) (Table 2). The prevalence of cannabis use was also higher among those in the youngest age group, with 7592 patients aged 18 to 29 years reporting cannabis use (31.0%; 95% CI 30.4%-31.6%), declining to 4200 among patients aged 60 years and older (8.5%; 95% CI, 8.3%-8.8%). Patients living in the most socioeconomically disadvantaged neighborhoods (ADI deciles 9-10) had the lowest prevalence of cannabis use (189 [13.8%; 95% CI, 12.0%-15.5%]) compared with those in the least disadvantaged neighborhoods (ADI deciles 1-2; 12 431 [17.4%; 95% CI, 17.1%-17.7%]).

**Table 2.** Prevalence of Cannabis Use and Cannabis Use Disorder Among Adult Primary Care Patients, January 2021 to May 2023, by Sociodemographic Characteristics

Characteristic	Cannabis use, past 3 mos^a^		Moderate to high risk for CUD^a^	
	No. (%) [95% CI]^b^	*P* value	No. (%) [95% CI]^b^	*P* value
Total	29 898 (17.0) [16.8-17.2]	NA	10 360 (34.7) [34.1-35.2]	NA
Age, y				
18-29	7592 (31.0) [30.4-31.6]	<.001	2946 (38.8) [37.7-39.9]	<.001
30-39	8417 (23.1) [22.7-23.6]		2904 (34.5) [33.5-35.5]	
40-49	5658 (16.9) [16.5-17.3]		1832 (32.4) [31.2-33.5]	
50-59	4031 (12.6) [12.2-12.9]		1227 (30.4) [29.0-31.8]	
≥60	4200 (8.5) [8.3-8.8]		1451 (34.6) [33.1-36.0]	
Sex				
Female	14 916 (14.7) [14.5-14.9]	<.001	4626 (31.0) [30.3-31.8]	<.001
Male	14 939 (20.0) [19.9-20.5]		5720 (38.3) [37.5-39.1]	
Sexual minority^c^				
Yes	3870 (33.1) [32.3-34.0]	<.001	1609 (41.6) [40.0-43.1]	<.001
No	23 389 (15.9) [15.8-16.1]		7803 (33.4) [32.8-34.0]	
Race and ethnicity				
American Indian or Alaska Native	61 (19.6) [15.1-24.0]	<.001	25 (41.0) [28.6-53.3]	.08
Asian	2667 (10.6) [10.2-10.9]		828 (31.1) [29.3-32.8]	
Black or African American	1386 (19.6) [18.7-20.6]		588 (42.4) [39.8-45.0]	
Hispanic	3691 (16.8) [16.3-17.3]		1409 (38.2) [36.6-39.7]	
Middle Eastern or North African	673 (14.4) [13.4-15.4]		250 (37.2) [33.5-40.9]	
Other^d^	9778 (19.3) [19.0-19.7]		3187 (32.6) [31.7-33.5]	
White	9628 (18.9)		3354 (34.8) [33.9-35.8]	
Employed				
Yes	22 583 (16.7) [16.5-16.9]	.05	7674 (34.0) [33.4-34.6]	<.001
No	3556 (16.1) [15.7-16.6]		1346 (37.9) [36.3-39.5]	
State ADI rank				
Decile 1-2	12 431 (17.4) [17.1-17.7]	<.001	3987 (32.1) [31.3-32.9]	<.001
Decile 3-4	5792 (17.6) [17.1-17.9]		2173 (37.5) [36.3-38.8]	
Decile 5-6	2667 (16.9) [16.3-17.4]		1035 (38.8) [37.0-40.7]	
Decile 7-8	979 (16.6) [15.6-17.5]		378 (38.6) [35.6-41.7]	
Decile 9-10	189 (13.8) [12.0-15.5]		74 (39.2) [33.2-46.1]	

Abbreviations: ADI, Area Deprivation Index; CUD, cannabis use disorder; NA, not applicable.

^a^ Sum may not equal to total due to missing data with missingness as follows: 0 for age; 148 (0.08%) for sex; 14 779 (8.4%) for race and ethnicity; 18 510 (10.5%) for employment; 17 300 (9.8%) for sexual identity; and 48 307 (27.5%) for state ADI rank.

^b^ Prevalence estimates represent row percentages.

^c^ Sexual minority includes those identifying as lesbian, gay, bisexual, or other sexual orientation.

^d^ Other race and ethnicity includes those who selected other, multiple races, or do not identify with a race.

Among patients who reported cannabis use, 10 360 (34.7%; 95% CI, 34.1%-35.2%) had ASSIST scores indicative of moderate to high risk for CUD (Table 2). Being at moderate to high risk for CUD was highest among those aged 18 to 29 years (2946 [38.8%; 95% CI, 37.7%-39.9%]) and lowest among those aged 50 to 59 years (1227 [30.4%; 95% CI, 29.0%-31.8%]). Furthermore, being at moderate to high risk for CUD was higher among patients living in the most socioeconomically disadvantaged neighborhoods (ADI deciles 9-10) compared with those in the least disadvantaged neighborhoods (ADI deciles 1-2) (74 [39.2%; 95% CI, 33.2%-46.1%] vs 3987 [32.1%; 95% CI, 31.3%-32.9%]).

### Frequency, Mode, and Reasons for Cannabis Use

Among 29 898 patients who reported cannabis use, 11 989 (40.1%) reported using only once or twice in the past 3 months, 4963 (16.6%) reported using monthly, 7325 (24.5%) reported using weekly, and the remaining 5621 (18.8%) reported using cannabis daily or almost daily. The most common modes of use included edibles (18 201 [61.6%]), smoking (15 256 [51.7%]), and vaporizing (8555 [29.0%]) (Figure). More than half the patients reported using only 1 mode (10 415 [54.4%]), with 5916 (30.9%) reporting 2 modes, 2221 (11.6%) reporting 3 modes, and 594 (3.1%) reporting 4 or more different modes of cannabis use. While no meaningful differences in mode of cannabis use were noted by race and ethnicity or employment status, mode of use varied by age and sex. For instance, a higher proportion of male patients compared with female patients reported smoking or vaping cannabis, while female patients were more likely to report use of edibles, applying to skin, or drinking cannabis (Figure; eTables 1 and 2 in Supplement 1).

**Figure.**
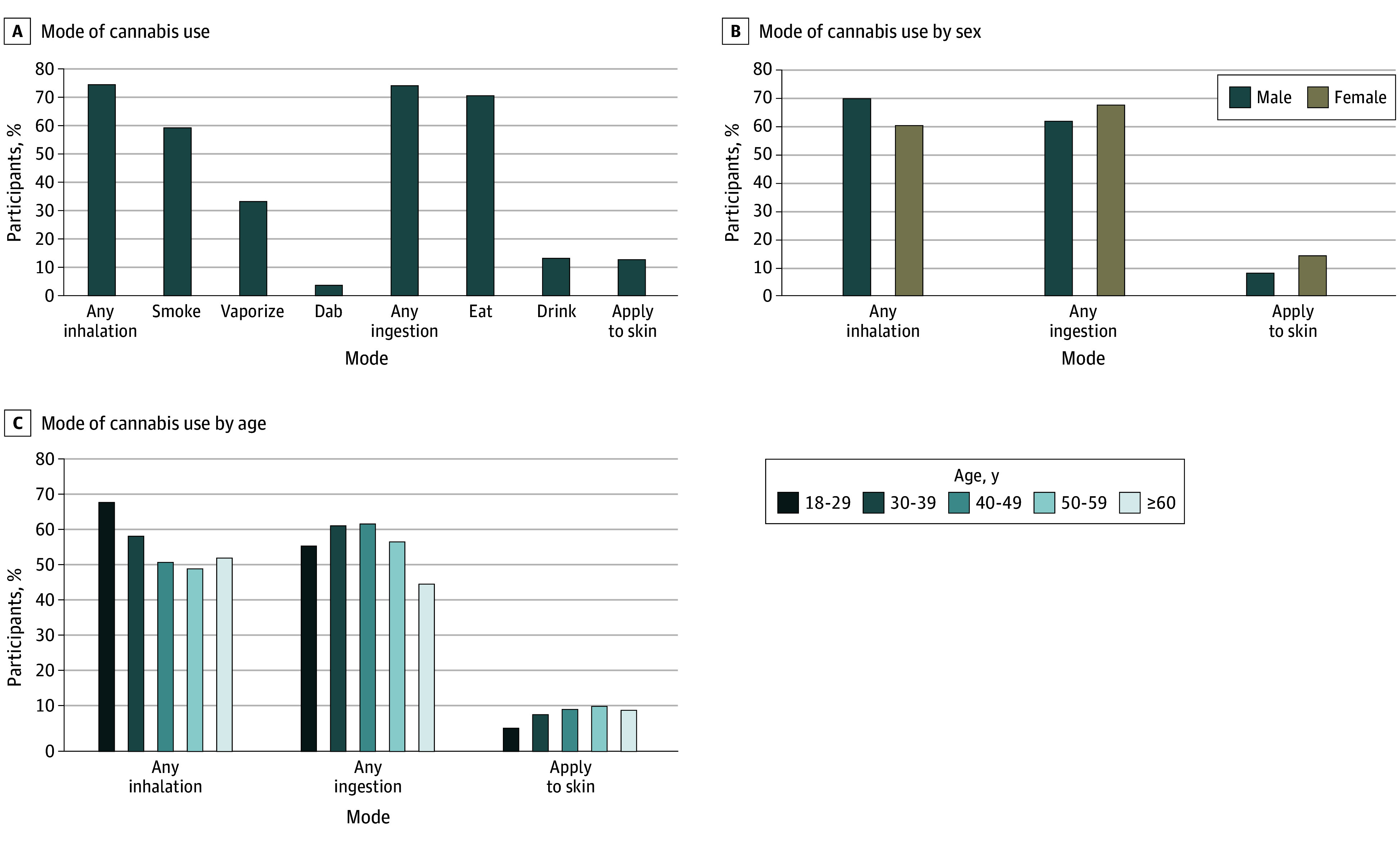
Mode of Cannabis Use Among 29 898 Adult Primary Care Patients Overall and by Age and Sex, January 2021 to May 2023

### Medical Cannabis Use and Reasons for Cannabis Use

Only 4375 patients (15.6%) reported using cannabis for strictly medical reasons, with 8734 (31.1%) reporting both medical and nonmedical reasons and the remaining 14 944 (53.3%) reporting only nonmedical reasons (Table 3). Nonetheless, 21 986 patients (75.7%) reported using cannabis to manage a range of symptoms, including pain, stress, and sleep (Table 3). The most common symptom for which cannabis use was reported was sleep (16 221 [56.0%]), followed by stress (14 542 [50.2%]), worry or anxiety (10 531 [36.3%]), and nonspecific pain (9196 [31.7%]). The median (IQR) number of symptoms managed with cannabis was 2 (1-4) and was higher among patients who were at moderate to high risk for CUD (4 [2-6] symptoms) compared with those at low risk for CUD (2 [1-4] symptoms) (*P* < .001). Likewise, the proportion of patients at moderate to high risk for CUD who reported using cannabis for any symptom was higher when compared with the overall sample (9233 [90.9%] vs 21 986 [75.7%]) (Table 3).

**Table 3.** Reasons for Cannabis Use Among Adult Primary Care Patients Who Reported Cannabis Use in the Past 3 Months, January 2021 to May 2023

Reason	Patients, No. (%)^a^	
	Cannabis use, past 3 mo (n = 29 898)	Moderate to high risk for CUD (n = 10 360)
Reasons for cannabis use, past 3 mo		
Only medical reasons	4375 (15.6)	1278 (12.8)
Only for nonmedical reasons	14 944 (53.3)	4009 (40.3)
Both medical and nonmedical reasons	8734 (31.1)	4669 (46.9)
No. of symptoms managed with cannabis, median (IQR)	2 (1-4)	4 (2-6)
Symptoms managed with cannabis, past 3 mo		
Any symptom	21 986 (75.7)	9233 (90.9)
Sleep	16 221 (56.0)	7246 (71.4)
Pain		
Any	10 591 (36.5)	5188 (51.1)
Pain, not specific	9196 (31.7)	4567 (45.0)
Muscle spasm	2916 (10.1)	1510 (14.9)
Headaches or migraines	3329 (11.5)	1913 (18.9)
Arthritis	1569 (5.4)	784 (7.7)
Mental health symptoms		
Any	16 100 (55.5)	7841 (77.2)
Stress	14 542 (50.2)	7290 (71.8)
Worry or anxiety	10 531 (36.3)	5653 (55.7)
Depression or sadness	5000 (17.3)	3451 (34.0)
Focus or concentration	2998 (10.4)	2108 (20.8)
PTSD	1407 (4.9)	971 (9.6)
Other symptoms		
Seizures or epilepsy	123 (0.4)	80 (0.8)
Nausea or vomiting	2820 (9.7)	1879 (18.5)
Appetite	3035 (10.5)	2230 (22.0)
Cancer	228 (0.8)	122 (1.2)
Glaucoma	126 (0.4)	61 (0.6)

Abbreviations: CUD, cannabis use disorder; PTSD, posttraumatic stress disorder.

^a^ Sum may not equal total because of missing data with missingness as follows: 1845 (6.2%) for reasons for cannabis use; 856 (2.9%) for symptoms managed with cannabis.

### Factors Associated With Cannabis Use and Risk of CUD

After adjusting for sex, race and ethnicity, and employment status, age remained independently associated with cannabis use, with those in the youngest age group (18-29 years) having more than 5 times the odds of cannabis use compared with aged 60 years and older (adjusted odds ratio [AOR], 5.2; 95% CI, 4.9-5.5) (Table 4). Patients who lived in the most economically disadvantaged neighborhoods (ADI decile 9-10) had decreased odds of cannabis use compared with those who lived in the least disadvantaged neighborhoods (ADI decile 1-2) (AOR, 0.7; 95% CI, 0.6-0.9). However, risk for CUD increased with level of neighborhood disadvantage and was higher among patients who lived in the most disadvantaged neighborhoods (AOR, 1.4; 95% CI, 1.1-1.9).

**Table 4.** Factors Associated With Cannabis Use and CUD Among Adult Primary Care Patients, January 2021 to May 2023

Factor	aOR (95% CI)	
	Cannabis use, past 3 mo	Moderate-high risk for CUD^a^
Age, y		
18-29	5.2 (4.9-5.5)	1.2 (1.0-1.3)
30-39	3.5 (3.3-3.7)	0.9 (0.8-1.0)
40-49	2.4 (2.3-2.5)	0.9 (0.8-1.0)
50-59	1.6 (1.6-1.7)	0.8 (0.7-0.9)
≥60	1 [Reference]	1 [Reference]
Sex		
Female	1 [Reference]	1 [Reference]
Male	1.6 (1.5-1.7)	1.5 (1.4-1.6)
Sexual minority^b^		
Yes	1.9 (1.8-2.0)	1.4 (1.3-1.6)
No	1 [Reference]	1 [Reference]
Race and ethnicity		
American Indian or Alaska Native	1.2 (0.8-1.7)	0.9 (0.4-1.9)
Asian	0.5 (0.4-0.5)	0.8 (0.7-0.9)
Black or African American	1.1 (1.0-1.2)	1.3 (1.1-1.5)
Hispanic	0.7 (0.6-0.7)	1.2 (1.1-1.3)
Middle Eastern or North African	0.6 (0.5-0.7)	0.9 (0.8-1.0)
Other^c^	1.0 (1.0-1.1)	0.9 (0.8-1.0)
White	1 [Reference]	1 [Reference]
Employed		
Yes	0.9 (0.9-1.0)	1.2 (1.1-1.4)
No	1 [Reference]	1 [Reference]
State ADI rank		
Decile 1-2	1 [Reference]	1 [Reference]
Decile 3-4	1.0 (0.9-1.0)	1.2 (0.1-1.4)
Decile 5-6	0.9 (0.8-0.9)	1.3 (1.1-1.4)
Decile 7-8	0.9 (0.8-0.9)	1.3 (1.1-1.5)
Decile 9-10	0.7 (0.6-0.9)	1.4 (1.1-1.9)

Abbreviations: aOR, adjusted odds ratio; CUD, cannabis use disorder; ADI, Area Deprivation Index.

^a^ Among those who reported cannabis use; based on Alcohol Substance Involvement Screening Test scores of 8 or greater.

^b^ Sexual minority includes those identifying as lesbian, gay, bisexual, or other sexual orientation.

^c^ Other race and ethnicity includes those who selected other, multiple races, or do not identify with a race.

## Discussion

Our study found that among the 74.8% of patients who completed cannabis screening, nearly 1 in 5 adult primary care patients reported recent cannabis use, among whom a third were at moderate to high risk for CUD. These rates are higher than those among adults in the general population; however, our results are comparable with the small number of recent studies that have estimated cannabis use within health care settings.^1,16,31,32^ We also found that while a minority of patients reported using cannabis for strictly medical reasons, most of those who reported cannabis use reported using it to manage a specific health symptom. This aligns with another study^29^ that found that this type of cannabis use is clinically underrecognized, and without specifically screening for medical cannabis use, clinicians may not ask and patients often do not disclose their use. As one of the first health systems to routinely ask about reasons for cannabis use, including health symptoms managed with cannabis, we provide a crucial point-of-care opportunity for clinicians to understand their patients’ risk for CUD given the association between reasons for cannabis use and the risk of disordered use.^32^

The data from this study show that the prevalence of cannabis use and the risk of disordered use were highest among male patients and younger adults. Comparable with findings from a recent study,^32^ more than a third of patients who reported cannabis use were at moderate to high risk for CUD (5.8% of the overall sample). This group could benefit from a primary care clinician–based brief intervention to prevent those at moderate risk for cannabis use disorders from developing more serious CUD and to evaluate and refer high-risk users for possible addiction treatment.^33,34,35^ Interestingly, while the prevalence of cannabis use was lowest among patients living in the most disadvantaged neighborhoods, risk for CUD was higher among this group. The influence of neighborhood on health outcomes has been well established; however, the influence of neighborhood on substance use and especially cannabis use is limited or conflicting.^36,37,38^ In this study, the underrepresentation of patients from the most disadvantaged neighborhoods prevents drawing strong conclusions on the specific links with risk of CUD.

Less than half the patients who used cannabis reported using it for medical reasons, even though the majority of patients reported cannabis use to manage a health-related symptom. Given these discrepant findings, it may be more useful for clinicians to ask patients what symptoms they are using cannabis for rather than relying on patient self-identification as a recreational or medical cannabis user. Moreover, primary care clinicians should take note that if patients are using cannabis for 4 or more symptoms they might be more likely to be at risk for CUD. Despite the commonplace use of cannabis to manage these symptoms, there is little evidence to guide clinicians on how to advise their patients regarding the benefits of cannabis for alleviating these symptoms.

Ingestion of cannabis was as common as inhalation among patients in this study. This is in contrast to studies among nonclinical populations in which inhaling cannabis exceeds ingestion.^39,40^ Ingestion modes in the form of edibles, such as brownies, cookies, candy, pills, or beverages, present a greater risk for poisoning or acute intoxication among patients, especially given that it may be more difficult to estimate the amount of cannabis that is ingested.^41,42^ Ingestion modes may also present more difficulty than inhaled modes for patients to titrate the cannabis dosage for symptom management, and given drastic increases in potency and lack of regulations, this may be of concern.^43^

### Limitations

The findings of this study should be interpreted in the context of several limitations. Screening for cannabis use was based on patient self-report. Although minimized by legalization of cannabis in California, patients may be reluctant to disclose their cannabis use, resulting in response bias. Beyond that, the symptoms for which cannabis use was reported were also based on self-report and not validated against diagnosed conditions in the EHR, which in turn limits our interpretation strictly to use of cannabis for symptom management rather than for clinically diagnosed conditions. Data for this study include screening conducted during the restriction phases of the COVID-19 pandemic, which may have resulted in higher rates of cannabis use. Furthermore, findings from this study are based on the sample of patients enrolled in a large health care system who came in for a primary care visit and thus might not be generalizable to all patients in the health system or to patients in other health systems, especially those where adult cannabis use is not legalized.

## Conclusions

In this study, 17.0% of primary care patients screened reported cannabis use, of whom 34.7% had ASSIST scores indicating increased risk of developing CUD. Most patients reported that they used cannabis to manage symptoms including stress and pain. Given the high rates of cannabis use and medical cannabis use that we found in this large urban health care system, it is essential that health care systems implement routine screening of all primary care patients. Integrating screening efforts to include information regarding cannabis use for symptom management could help enhance the identification and documentation of medical cannabis usage, particularly in the health care context.

## Notes

Supplement 1. **eAppendix.** Tobacco and Cannabis Questionnaire (TCQ) **eTable 1.** Inhaled Modes of Cannabis Use Among Patients Who Reported Cannabis Use in the Past 3 Months, by Demographic Factors **eTable 2.** Ingestion Modes and Skin Application of Cannabis Use Among Patients Who Reported Cannabis Use in the Past 3 Months, by Demographic Factors

Supplement 2.
Data Sharing Statement
