# Supplementary material for: Cannabis Use Reported by Patients Receiving Primary Care in a Large Health System
Source: JAMA Netw Open. 2024 Jun 5;7(6):e2414809. doi: 10.1001/jamanetworkopen.2024.14809 (PMC11154156; doi:10.1001/jamanetworkopen.2024.14809)
Supplement: Supplement 1. — eAppendix. Tobacco and Cannabis Questionnaire (TCQ) eTable 1. Inhaled Modes of Cannabis Use Among Patients Who Reported Cannabis Use in the Past 3 Months, by Demographic Factors eTable 2. Ingestion Modes and Skin Application of Cannabis Use Among Patients Who Reported Cannabis Use in the Past 3 Months, by Demographic Factors [file jamanetwopen-e2414809-s001.pdf]

Supplemental Online Content

Gelberg L, Beck D, Koerber J, et al. Cannabis use reported by patients receiving primary care in a large health system. *JAMA Netw Open.* 2024;7(6):e2414809. doi:10.1001/jamanetworkopen.2024.14809

eAppendix. Tobacco and Cannabis Questionnaire (TCQ)

eTable 1. Inhaled Modes of Cannabis Use Among Patients Who Reported Cannabis Use in the Past 3 Months, by Demographic Factors

eTable 2. Ingestion Modes and Skin Application of Cannabis Use Among Patients Who Reported Cannabis Use in the Past 3 Months, by Demographic Factors

This supplemental material has been provided by the authors to give readers additional information about their work.

© 2024 Gelberg L et al. *JAMA Network Open.*

eAppendix. Tobacco and Cannabis Questionnaire (TCQ)

The following questions are about your experiences with tobacco and cannabis (marijuana, weed, pot, hash, grass, etc.) products. Some of the questions ask about your experiences with these substances in the last 3 months and some about your experiences with these substances over your whole life. The substances can be smoked, swallowed, snorted, inhaled, injected, taken in pill form, or applied to the skin.

For cannabis use, **do not include CBD only products** such as in *lotions, ointment, and CBD-only products (such as hemp)*

Please note that this questionnaire is sent to all UCLA patients

ASSIST1 (A1)

**In your life, which of the following substances have you ever used ? (non-medical use only)**	**No**	**Yes**
a) Tobacco products (cigarettes, e-cigarettes, JUUL, vape pen, hookah, chewing tobacco, cigars, etc.)	0	3
b) Cannabis (marijuana, pot, grass, hash, weed, etc.)	0	3

ASSIST2 (A2)

**In the past 3 months, how often have you used the following substances?**	**Never**	**Once or Twice**	**Monthly**	**Weekly**	**Daily or Almost Daily**
c) Tobacco products (cigarettes, e-cigarettes, JUUL, vape pen, hookah, chewing tobacco, cigars, etc.)	0	2	3	4	6
d) Cannabis (marijuana, pot, grass, hash, weed, etc.)	0	2	3	4	6

ASSIST3 (A3)

**In the past 3 months, how often have you had a strong desire to use the following substance(s):**	**Never**	**Once or Twice**	**Monthly**	**Weekly**	**Daily or Almost Daily**
a) Tobacco products (cigarettes, e-cigarettes, JUUL, vape pen, hookah, chewing tobacco, cigars, etc.)	0	3	4	5	6
b) Cannabis (marijuana, pot, grass, hash, weed, etc.)	0	3	4	5	6

© 2024 Gelberg L et al. *JAMA Network Open.*

ASSIST4 (A4)

**During the past 3 months, how often has your use of the following substance(s) led to health, social, legal or financial problems?**	**Never**	**Once or Twice**	**Monthly**	**Weekly**	**Daily or Almost Daily**
a) Tobacco products (cigarettes, e-cigarettes, JUUL, vape pen, hookah, chewing tobacco, cigars, etc.)	0	4	5	6	7
b) Cannabis (marijuana, pot, grass, hash, weed, etc.)	0	4	5	6	7

ASSIST5 (A5)

**During the past 3 months, how often have you failed to do what was normally expected of you because of your use of the following substance(s)?**	**Never**	**Once or Twice**	**Monthly**	**Weekly**	**Daily or Almost Daily**
a) Tobacco products (cigarettes, e-cigarettes, JUUL, vape pen, hookah, chewing tobacco, cigars, etc.)	-	-	-	-	-
b) Cannabis (marijuana, pot, grass, hash, weed, etc.)	0	5	6	7	8

ASSIST6 (A6)

**Has a friend or relative or anyone else ever expressed concern about your use of the following substance(s)?**	**No, Never**	**Yes, but not in the past 3 months**	**Yes, in the past 3 months**
a) Tobacco products (cigarettes, e-cigarettes, JUUL, vape pen, hookah, chewing tobacco, cigars, etc.)	0	3	6
b) Cannabis (marijuana, pot, grass, hash, weed, etc.)	0	3	6

ASSIST7 (A7)

**Have you ever tried to control, cut down or stop using but failed in your attempts for the following substance(s)?**	**No, Never**	**Yes, but not in the past 3 months**	**Yes, in the past 3 months**
a) Tobacco products (cigarettes, e-cigarettes, JUUL, vape pen, hookah, chewing tobacco, cigars, etc.)	0	3	6
b) Cannabis (marijuana, pot, grass, hash, weed, etc.)	0	3	6

© 2024 Gelberg L et al. *JAMA Network Open.*

TOBACCO USERS ONLY

T1.

**In the past 3 months, how often did you use cigarettes ONLY?**	**Never**	**Once or Twice**	**Monthly**	**Weekly**	**Daily or Almost Daily**
	0	2	3	4	6

T2.

**In the past 3 months, how often did you use e-cigarettes (such as JUUL, vape pen)?**	**Never**	**Once or Twice**	**Monthly**	**Weekly**	**Daily or Almost Daily**
	0	2	3	4	6

T3.

**In the past 3 months, how often did you use hookah water pipe?**	**Never**	**Once or Twice**	**Monthly**	**Weekly**	**Daily or Almost Daily**
	0	2	3	4	6

T4.

**In the past 3 months, how often did you use chewing tobacco, snuff or dip?**	**Never**	**Once or Twice**	**Monthly**	**Weekly**	**Daily or Almost Daily**
	0	2	3	4	6

T5. For how many years have you smoked cigarettes?

Number of Years: ___ ___

T6. On average, how many cigarettes do you smoke per day? There are 20 cigarettes in a pack.

Number of Cigarettes: ___ ___

© 2024 Gelberg L et al. *JAMA Network Open.*

CANNABIS USERS ONLY

M1. During the past 3 months, how did you use cannabis? **(do not include CBD only products)** Please select all that apply. Did you…

1. Smoke it (in a joint, bong, blunt, spliff or pipe)
2. Eat it (in brownies, cakes, cookies, or candy, pills)
3. Drink it (in a tincture, tea, cola, or alcohol)
4. Vaporize it (hash oil in an e-cigarette-like vaporizer or another vaporizing device)
5. Dab it (using waxes or concentrates in a dab rig or other device)
6. Apply it to skin (lotion, ointment, patch or salve)

M2. On a typical day that you use cannabis, how many times per day do you use it**? (do not include CBD only products)**

1. 1
2. 2-3
3. 4-5
4. 6-9
5. 10 or more

M3. During the past 3 months, when you used cannabis (**do not include CBD only products**) was it:

1. Only for medical reasons
2. Only for non-medical reasons
3. Both medical and non-medical reasons

M4. During the past 3 months, have you used cannabis to help you manage any of the following (do not include **CBD only** products)?

1. Pain
2. Muscle Spasm
3. Seizures/epilepsy
4. Nausea or vomiting
5. Sleep
6. Stress
7. Appetite
8. Worry or anxiety
9. Depression or sadness

© 2024 Gelberg L et al. *JAMA Network Open.*

10. Focus or concentration
11. Headaches/migraines
12. Post-traumatic Stress Disorder (PTSD)
13. Acquired Immune Deficiency Syndrome (AIDS)
14. Arthritis
15. Cancer
16. Glaucoma
17. Other reasons for cannabis use:

© 2024 Gelberg L et al. *JAMA Network Open.*

CBD ONLY USE

C1.

**In the past 3 months, how often have you used:**	**Never**	**Once or Twice**	**Monthly**	**Weekly**	**Daily or Almost [illegible]**
e) **CBD only** *lotions, ointment, and other CBD-only products (such as hemp)?*	0	2	3	4	6

SECOND HAND EXPOSURE RISK

S1. In the past 7 days, were you exposed to secondhand tobacco or cannabis smoke where you live, someone else's home, work, or in a car?

1. Only Tobacco exposure
2. Only Cannabis exposure
3. Both Tobacco and Cannabis exposure
4. No Exposure to tobacco or cannabis

© 2024 Gelberg L et al. *JAMA Network Open.*

eTable 1. Inhaled Modes of Cannabis Use Among Patients Who Reported Cannabis Use in the Past 3 Months, by Demographic Factors

	Inhalation							
	Total		Smoke		Vaporize		Dab	
	n (%)	p value	n (%)	p value	n (%)	p value	n (%)	p value
Total	19,146 (65.0)	--	15,256 (51.7)	--	8,555 (29.0)	--	956 (3.2)	
Age		<.001		<.001		<.001		<.001
18-29	5,752 (76.5)		4,880 (64.9)		2,731 (36.3)		572 (7.6)	
30-39	5,057 (66.1)		4,380 (51.7)		2,676 (32.1)		203 (2.4)	
40-49	3,251 (58.1)		2,378 (42.5)		1,594 (28.5)		98 (1.8)	
50-59	2,216 (11.6)		1,717 (43.4)		850 (21.5)		46 (1.2)	
60+	2,420 (59.4)		1,973 (48.4)		704 (17.1)		37 (0.9)	
Sex		<.001		<.001		<.001		0.032
Female	8,884 (60.3)		7,095 (48.1)		3,932 (26.6)		445 (3.0)	
Male	10,235 (69.7)		8,139 (55.4)		4,611 (31.3)		509 (3.5)	
Race/Ethnicity		<.001		<.001		0.334		0.010
American Indian/Alaska Native	34 (55.7)		29 (47.5)		11 (18.0)		0 (0.0)	
Asian	1,631 (61.9)		1,208 (45.8)		820 (31.1)		68 (2.3)	
Black/African American	999 (73.4)		874 (64.0)		332 (24.3)		47 (3.4)	
Hispanic	2,594 (70.9)		2,175 (59.4)		1,126 (30.7)		200 (5.5)	
Middle Eastern/North African	449 (68.7)		362 (55.3)		205 (31.4)		15 (2.3)	
Other	5,994 (62.1)		4,683 (48.5)		2,762 (28.6)		282 (2.9)	
White	6,141 (64.7)		4,858 (51.2)		2,761 (29.0)		271 (2.9)	

Note: Mode of cannabis use was missing for 424 (1.4%) of patients

© 2024 Gelberg L et al. *JAMA Network Open.*

eTable 2. Ingestion Modes and Skin Application of Cannabis Use Among Patients Who Reported Cannabis Use in the Past 3 Months, by Demographic Factors

	Ingestion						Apply to Skin	
	Total		Eat		Drink			
	n (%)	p value	n (%)	p value	n (%)	p value	n (%)	p value
Total	19,090 (64.7)		18,201 (61.6)		3,395 (11.5)		3,261 (11.1)	
Age		<.001		<.001		<.001		<.001
18-29	4,744 (63.1)		4,558 (60.6)		861 (11.5)		534 (7.1)	
30-39	5,780 (69.3)		5,548 (66.5)		1,169 (14.0)		943 (11.3)	
40-49	3,910 (69.9)		3,715 (66.4)		706 (12.6)		723 (12.9)	
50-59	2,549 (64.4)		2,405 (60.7)		382 (9.7)		545 (13.8)	
60+	2,107 (51.4)		1,975 (48.1)		277 (6.8)		516 (12.6)	
Sex		<.001		<.001		<.001		<.001
Female	9,963 (67.5)		9,437 (63.9)		1,889 (12.8)		2,079 (14.1)	
Male	9,099 (61.9)		8,736 (59.3)		1,502 (10.2)		1,179 (8.0)	
Race/Ethnicity		0.885		0.415		0.589		0.468
American Indian/Alaska Native	40 (66.7)		38 (63.3)		61 (9.8)		8 (13.1)	
Asian	1,839 (69.7)		1,775 (67.3)		273 (10.4)		202 (7.7)	
Black/African American	796 (58.2)		756 (55.3)		188 (13.8)		172 (12.6)	
Hispanic	2,243 (61.3)		2,152 (58.8)		416 (11.4)		481 (13.1)	
Middle Eastern/North African	373 (57.1)		360 (55.1)		55 (8.4)		58 (8.8)	
Other	6,511 (67.4)		6,193 (64.0)		1,167 (12.1)		1,065 (11.0)	
White	6,101 (64.2)		5,798 (61.0)		1,072 (11.3)		993 (10.5)	

Note: Mode of cannabis use was missing for 424 (1.4%) of patients

© 2024 Gelberg L et al. *JAMA Network Open.*
