# Supplementary material for: Cannabis Use Reported by Patients Receiving Primary Care in a Large Health System
Source: JAMA Netw Open. 2024 Jun 5;7(6):e2414809. doi: 10.1001/jamanetworkopen.2024.14809 (PMC11154156; doi:10.1001/jamanetworkopen.2024.14809)
Supplement: Supplement 2. — Data Sharing Statement [file jamanetwopen-e2414809-s002.pdf]

Data Sharing Statement

Gelberg. Cannabis Use Reported by Patients Receiving Primary Care in a Large Health System. *JAMA Netw Open*. Published June 05, 2024.
doi:10.1001/jamanetworkopen.2024.14809

Data
Data available: No

Additional Information
Explanation for why data not available: We used electronic health data with PHI/PII which restricts our ability to share the data
